# Microbial communities in pesticide-contaminated soils in Kyrgyzstan and bioremediation possibilities

**DOI:** 10.1007/s11356-017-0048-5

**Published:** 2017-09-07

**Authors:** Tinatin Doolotkeldieva, Maxabat Konurbaeva, Saykal Bobusheva

**Affiliations:** Plant Protection Department, Kyrgyz-Turkish Manas University, 56 Prospect Mira, Bishkek, Kyrgyzstan

**Keywords:** Obsolete pesticide dump, Contaminated soil, Microbial consortia, Bacterial populations with P450 cytochrome genes, active bacteria for aerobic bioremediation of pesticides

## Abstract

In Kyrgyzstan, many former storehouses and dump sites for obsolete pesticides exist. In 2009/2010, an inventory and assessment of these sites including risks of environmental hazard has been conducted by FAO and the World Bank. Monitoring revealed high concentration of pesticides listed as persistent organic pollutants (POPs). The purpose of this research was to study the microbial structural complexes of the pesticide-contaminated soils in these dumping zones, and to search for and select microorganism’s destructors with cytochrome P450 genes for pesticide degradation. Culture-dependent and culture-independent approaches were used to determine the taxonomic composition of these bacterial communities. The universal primer set for the 16S ribosomal RNA (rRNA) gene and the specific primer set P450R were used to amplify the cytochrome P450 hydroxylase gene. In soils from Suzak A and B and soils from Balykchy dumping sites, the bacteria from the *Actinobacteria* phylum (*Micrococcus* genus) were dominant. These bacteria made up 32–47% of the indigenous local microflora; bacteria species from the *Pseudomonas* genus (*Gammaproteobacteria* phylum) made up 23% in Suzak, 12% in Balykchy soils. *Bacillus* species from the *Firmicutes* phylum were found only in Suzak soils. The 16S rRNA analyses and the specific primer set P450R have revealed bacteria with cytochrome genes which are directly involved in the degradation process of organic carbon compounds. Experiments were carried out to help select active degraders from the bacterial populations isolated and used to degrade Aldrin in laboratory. Active bacterial strains from the *Pseudomonas fluorescens* and *Bacillus polymyxa* population were selected which demonstrated high rates of degradation activity on Aldrin.

## Introduction

Pesticides are widely used in agricultural production to prevent or reduce losses caused by pests. Over 1 billion pounds of pesticides are used in the USA each year, and approximately 5.6 billion pounds are used worldwide (United States Environmental Protection Agency 2011). Estimates concerning the number of obsolete stocks around the world range from 300,000 to 500,000 t (FAO 2014, Pieterse et al. 2015).

Classes of organic pesticides include organochlorines, organophosphates, organometallic compounds, pyrethroids, and carbamates, among others (Gilden et al. 2010). However, their chemical structures, along with their incorrect preparation, application, and storage, may pose a serious toxicity risk to other organisms, both environmental and human (Fantke et al. 2012; Pieterse et al. 2015; Torres et al. 2013). In the present age, more than 500 different formulations of pesticides are used, mainly in agricultural tricks. These formulations are in general artificially synthesized substances that are non-biodegradable and enhance environmental toxicity. These non-biodegradable compounds persist in agricultural fields after application. About three million people are intoxicated per annum as a result of pesticide usage, as reported by the World Health Organization (WHO) (Veiga et al. 2006).

Kyrgyzstan is a unique country with a rich natural and agricultural biodiversity in almost every region. However, there is a large legacy of former storehouses and dumping zones for obsolete pesticides and wastes from pesticide production, as is often observed in former Soviet countries (Toichuev et al. 2017a, b; Vijgen et al. 2013; Amirova and Weber 2015) but also occurs in other regions (Jit et al. 2010; Wycisk et al. 2013). In recent years in Kyrgyzstan (2009, 2010), an inventory and assessment of these dumping zones, and the risk of environmental hazard that they pose, has been performed by international organizations (FAO, World Bank) and related international experts. In the territory of Kyrgyzstan, 50 storage facilities of obsolete pesticides exist, which store about 5000 t of these hazardous chemicals. In many places, the local populations try to dig out obsolete pesticides for reuse. They pose a serious threat to the people living there, to livestock, and to the environment (World Bank 2010; Toichuev et al. 2017b).

The pesticide level in the environment determines the dose and time at which an organism is exposed. Due to the mobility and persistence of these semi-volatile compounds, they can represent a hazard for the wider environment and can travel to remote locations. Hence, their persistence in the environment leads to a risk for life. Pesticide persistence in the environment is caused by either the physico-chemical properties of the pesticide or the lack of organisms able to degrade it. Degradation caused by organisms (biodegradation) could help decrease considerably pesticide persistence in the environment (Velázquez-Fernández et al. 2012; Lal et al. 2010).

Microorganisms are vital for the bioremediation of pesticides. The phenomenon of biotransformation is very common and is sometimes very essential for the survival of microorganisms, and is responsible for the biodegradation of applied pesticides. There is a natural balance between microbial evolution and bioremediation (Hodgson et al. 1993).

The microbial degradation of xenobiotics, a process known as bioremediation, is a cost-effective method of removing pollutants from the environment. Biodegradation involves biological reactions that modify the chemical structure of the compound, which in turn implies a decrease in toxicity. When pesticide degradation occurs, it usually involves more than one microorganism. Each microorganism contributes to biodegradation reactions in pesticides, but no example of mineralization by a single strain has been described. Bacteria have been used extensively for bioremediation purposes (Sylvia et al. 2005). The bacterial classes gamma-proteobacteria (v.gr. *Pseudomonas*, *Aerobacter*, *Acinetobacter*, *Moraxella*, *Plesiomonas*), beta-proteobacteria (v.gr. *Burkholderia*, *Neisseria*), alpha-proteobacteria (v.gr.: *Sphingomonas*), *Actinobacteria* (*Micrococcus*), and *Flavobacteria* (*Flavobacterium*) are considered to be active microbiodegraders (Geetha and Fulekar 2008; Matsumoto et al. 2008; Mamta and Khursheed 2015). Five bacterial genera were able to degrade endosulfan, including *Klebsiella*, *Acinetobacter*, *Alcaligenes*, *Flavobacterium*, and *Bacillus*. During biodegradation, metabolites of endosulfan diol, endosulfan lactone, and endosulfan ether were also produced, but these had lesser toxicity compared with the original compound (i.e., endosulfan) (Kafilzadeh et al. 2015).

Recently, *Bacterium raoultella* sp. has also been found to degrade pesticides. The complete biodegradation of the pesticide involves the oxidation of the parent compound, resulting in carbon dioxide and water, which provide energy to microbes. For soil in which the innate microbial population is not able to manage pesticides, the external addition of pesticide-degrading microflora is recommended. Degradation of pesticides by microbes depends not only on the enzyme system but also on conditions like temperature, pH, and the nutrients available in the soil. Some of the pesticides are easily degraded; however, some are recalcitrant because of the presence of anionic species in the compound. Besides organophosphorus compounds, the neonicotinoids are degraded by the *Pseudomonas* species (Uqab et al. 2016; Javaid et al. 2016).

The degradation of pesticides results in the production of carbon dioxide (CO_2_) and water (H_2_O) through the oxidation of parent compounds. The bacterium involved in the degradation process takes energy from these degradation products. The efficiency of the degradation process depends upon optimum atmospheric conditions, including temperature, soil pH, and moisture content. The modification of different bacterial specimens via genetic mutation also enhances the effectiveness of applied microbes. The biodegradable removal of pesticides has positive effects on the fertility of agricultural soil (Javaid et al. 2016).

Bacterial biodegradation can take place in anaerobic or aerobic conditions. Although different enzymes are active under each respective condition, it seems that both aerobic and anaerobic degradations need to happen in order to achieve mineralization (Langerhoff et al. 2001). It seems that anaerobic metabolism is more useful for dechlorination (Barragán-Huerta et al. 2007; Baczynski et al. 2010) while aerobic metabolism results in a cleavage in aromatic or aliphatic cyclic metabolites. The higher persistence of organochlorine in aerobic conditions (Singh et al. 1999) compared to anaerobic conditions might be caused by the absence of enzymes, or more likely by oxidative damage following organochlorine metabolism. The removal of heteroatoms (like halogens) or heteroatom-containing groups is frequently among the first steps in biodegradation. These steps are catalyzed by monooxygenases, dioxygenases, or peroxidases (Singh et al. 1999; Wackett 1995), which in aerobic conditions could generate large quantities of free radicals. Thus, anaerobic conditions are more effective for the biodegradation of organochlorine pesticides, while aerobic conditions are better for biodegrading the hydrocarbon metabolites from these pesticides (In situ treatment technologies for contaminated soil 2006). In spite of such requirements, some examples of organochlorine pesticide bioremediation have been accomplished in situ (Langerhoff et al. 2001; Qureshi et al. 2009). Among oxidoreductases, the most frequent are monooxygenases like cytochrome P450, dioxygenases, peroxidases, and oxidases; hydrolases like A-esterase are involved in biodegradation pathways. Cytochrome P450 (CYP) can catalyze reactions of oxidation, reduction, or the oxidative breakdown of xenobiotics (Lamb et al. 2009; Yang et al. 2010; Cools et al. 2011; Leitao 2009; Pelkonen and Raunio 1997; Gonzalez and Lee 1996; Bernhardt and Urlacher 2014).

The main purpose of this research is to study the microbial structural complexes of pesticide-contaminated soils in these dumping zones, and to search for and select microorganism destructors with cytochrome P450 genes for pesticide degradation.

## Materials and methods

### Environmental samples

Persistent organic pollutants (POPs) are persistent in the environment, bioaccumulative and/or toxic, and therefore require analytical methods that are sensitive enough to meet the low detection limits needed for the protection of the environment and human health. A variety of techniques, procedures, and instruments can be used which are well suited for different scenarios (Megson et al. 2016).

Environmental soil samples were collected from sites located around three dumping plots (Suzak A, Suzak B, and Balykchy) that have long been exposed to pesticide contamination (Table 2). To collect soil samples from contaminated sites, standard methods of soil microbiology (Methods of Soil Microbiology and Biochemistry) and specific methods (Wang et al. 2012; Mudge 2008; Morrison and Murphy 2006) were used. Specifically, the samples were collected using a stainless steel handheld corer. The first two cores were always discarded. Three cores (0e5 cm), taken over an area of 100 m^2^, were bulked together to form one sample. The samples were wrapped in aluminum foil twice and sealed in two plastic bags to minimize the possibility for contamination. The samples were clearly labeled and stored in containers appropriate for the analysis being undertaken. All samples were transported to the laboratory as soon as possible (preferably within 24 h) with appropriate chain of custody documentation (Megson et al. 2016). Upon receipt, the samples were stored at 20 °C until conduction of microbiological and chemical analyses.

The same types of soil, taken from both natural and virgin plots, were used as uncontaminated soil samples. The distance from contaminated soil was 100–120 km.

The soil was air-dried, ground, and passed through a sieve with 2-mm pores, before being stored in sealed containers at room temperature. The organic carbon, cation exchange capacity, and other physico-chemical parameters of the soil samples were analyzed (Table 2). The soil samples were suspended in distilled water (1:4 *w*/*v*), and the particles were allowed to settle. The pH of the suspension was determined using a pH meter (Thermo Scientific, Orion Laboratory Products). Electrical conductivity of the soil was determined in the filtrate of the water extract using a conductivity meter. The organic carbon (OC) content was determined by adopting the chromic acid wet digestion method according to the standard procedure of Walkley and Black, using a diphenylamine indicator (Walkley and Black 1934). Available potassium content in the soil was determined by using turbidimetric methods; calcium was determined by titration with a standard KMnO_4_ solution, and magnesium was determined via precipitation in an alkaline medium as magnesium ammonium phosphate. The carbonate in the soil was determined by the rapid titration method using a bromothymol blue indicator (Velmurugan et al. 2012). Soil samples from the uncontaminated and contaminated sites served as an inoculum source for the enrichment cultures in subsequent experiments.

### Analysis of contaminated soils for pesticides

Soil samples taken from the contaminated sites were analyzed for their pesticide concentration at the Chemical Laboratory of the ‘ILIM’ Ltd. Scientific Production Association, Bishkek, Kyrgyzstan (Chromatography, Master GC). Finally, an acetone/hexane-based extract was made, after which cleanup was performed using Florisil (U.S. Silica Co.). The purified extracts were analyzed by capillary gas chromatography (GC) coupled to a mass spectrometry (MS; either single-quad MS or triple-quad MS-MS). Compound identifications were based on retention times and their qualifier and target ions. Quantifications were based on peak size. Chemical analysis for the quantification of targeted POPs (presented in Table 2) was performed according to methods compliant with NEN-6980 of the Netherlands Standardization Institute (Netherlands Standardization Institute NEN 2010).

#### Isolation of pesticide-degrading bacteria

Soil samples obtained from the surface horizon of polluted places with pesticides around the burial Suzak A, Suzak B, and Balykchy were used for isolation of bacteria. Soil samples were collected by scraping surface material with a sterile spatula and then obtaining approximately 100 g samples from 2 to 10 cm below the surface. Samples were then stored at 4 °C until use. To isolate bacteria species from the soil, soil samples were analyzed using the acetate selection protocol of Travers et al. and the methods of soil microbiology and biochemistry (Methods of Soil Microbiology and Biochemistry 1991) with some modifications. Samples of 10 g were prepared from each soil sample and ground in a sterile porcelain mortar for 5 min in aseptic conditions. After grinding, the soil sample was washed in sterile water. Ten milliliters of Luria-Bertani broth, 1 g from each soil sample, was added and buffered with sodium acetate (0.25 M, pH 6.8) in a 125-ml flask. The broth was incubated in a shaker at 200 rpm for 4 h at 30 °C. A 1-ml aliquot was spread on nutrient agar plates (NA), and incubated at 30 °C for 48–72 h. The colonies were subcultured on new NA plates until pure cultures were obtained, and they were kept at 4 °C for further identification.

#### Phenotypic characterization of pesticide-degrading bacteria strains

Gram staining, colony shape, and bacteria movement were analyzed. The isolated bacterial cultures were studied for their ability to grow on meat-peptone broth (MPB), meat-peptone agar (MPA), oxidative-fermentative (OF), and catalase. The conventional tests were performed, such as protein hydrolysis, including reduction of nitrates to nitrites, reduction of nitrates to nitrogen, indole production (tryptophane), fermentation (glucose), arginine dihydrolase, gelatin hydrolysis, and the urea breath test. Twelve assimilation tests were also performed with substrates such as glucose, arabinose, mannose, mannitol, n-acetyl-glucosamine, maltose, sorbitol, dulcitol, potassium gluconate, capric acid, adipic acid, malate, trisodium citrate, and phenylacetic acid. Fluorescent, diffusible pigments, and growth at 4, 27, and 37 °C were also determined. Phenotypic and biochemical characteristics of the isolates were established according to the determinants (Bergey’s Manual of Determinative Bacteriology 2006). Isolated bacteria were grouped on the basis of their morphological, biochemical, and physiological characteristics.

### Extraction of total DNA and DNA extraction from pure cultures

DNA was extracted from the enrichment cultures during the active phase of microbial growth, using the UltraClean™ Soil DNA Isolation Kit (Mo Bio Laboratories, Carlsbad, CA) and an alternative protocol developed by the Mo Bio Laboratories. To process soil samples, 5 g of soil was mixed with 10 to 30 ml of phosphate-buffered saline (PBS) to create homogenized slurry. Samples were mixed for 1 h at room temperature and then centrifuged for 5 min at 123×*g*. The supernatant was removed and centrifuged at 20,000×*g* for 15 min. The supernatant was then carefully discarded, and the pellet was re-suspended in 1 ml of PBS. In order to extract DNA, 700 μl of the re-suspended soil extraction pellet was processed. The purified bacteria were incubated in MPM medium for 2 days at 25 °C. Cells were harvested at the early exponential growth phase, and their DNA was then extracted by the alternative protocol of the Mo Bio Laboratories. Successful DNA extraction was determined by agarose gel electrophoresis (1.0% agarose).

Amplification was performed with a MultiGene Thermal Cycler (TC9600-G/TC, Labnet International), using a 25-μl mixture containing 15 μl of PCR Master Mix (*Taq* DNA polymerase, MgCl_2_, deoxyribonucleotide triphosphate, and reaction buffer), 2 μl of each primer, 1 μl of template DNA, and 1 μl of H_2_O. The amplification program was used as the following: 94 °C for 5 min, 35 cycles of 94 °C for 30 s, 55 °C for 30 s, 72 °C for 60 s, and 72 °C for 7 min. PCR products were electrophoresed in a 1.0% agarose gel and visualized using the BioDoc-It™ Imaging Systems (Ultra-Violet Products Ltd) after ethidium bromide staining. To control contamination, we used a negative control reaction and sterile water was added as a matrix.

Almost full-length fragments of 16Sp RNA genes were amplified using the primers 16S-27F and 16S-907R. Fragments of genes encoding the subunits of alkane monooxygenases were amplified using specific sets of primers. The primer set alkB-F and alkB-R was used to amplify the alkane hydroxylase, and P450R was used to amplify the cytochrome P450 alkane hydroxylase.

Sequence analysis was performed by the Macrogen Company (10F World Meridian Center, #60-24 Gasan-dong, Geumcheon-gu, Seoul, Korea, 153-023), and sequences were edited with Applied Biosystems 3730XL sequencers. Only sequences with more than 700 nucleotides were used for diversity analyses. The phylogenetic relatedness among different sites was determined using the cluster environment. The 16S ribosomal RNA (rRNA) gene sequences were deposited in the GenBank and DB of the National Center for Biotechnology Information nucleotide sequence databases.

### Biodegradation experiments

A mineral medium was prepared containing (NH_4_)_2_SO_4_ –1, 0, K_2_HPO_4−_0, 8, KH_2_PO_4−_0, 2, MgSO_4_*7H_2_O – 0, 2, CaCl_2_ *2H_2_O – 0, 1, FeCl_3_*6H_2_O – 0, 05, (NH_4_) Mo_4_O_2_*4H_2_O – 0, 01 and water (1000 ml, pH: 7.0). In this medium, microbial cultures were incubated with various concentrations (0.2, 0.5, and 1.0 mg) of the pesticide Aldrin (Table 1). Aldrin was selected as an organochlorine in order to test the degradation capability. The Aldrin sample in pure form was obtained from private agrochemicals companies from China in Bishkek, Kyrgyzstan.

**Table 1:** The variants of experiments to determine the ability of single bacterial culture and their association for biodegradation of obsolete pesticide, as Aldrin

Species of bacterial culture, in doses of 2 × 10^8^ CFU (colony-forming units) ml^−1^	Concentrations of Aldrin, in mg/per 50 ml of mineral medium	Incubation time of bacterial culture with pesticide, in days
*Pseudomonas fluorescens*	0.2 mg	2, 4, 6, and 12
*Pseudomonas fluorescens*	0.5 mg	2, 4, 6, and 12
*Pseudomonas fluorescens*	1.0 mg	2, 4, 6, and 12
*Micrococcus* sp.	0.2 mg	2, 4, 6, and 12
*Micrococcus* sp.	0.5 mg	2, 4, 6, and 12
*Micrococcus* sp.	1.0 mg	2, 4, 6, and 12
*Flavobacterium* sp.	0.2 mg	2, 4, 6, and 12
*Flavobacterium* sp.	0.5 mg	2, 4, 6, and 12
*Flavobacterium* sp.	1.0 mg	2, 4, 6, and 12
*Bacillus* sp.	0.2 mg	2, 4, 6, and 12
*Bacillus* sp.	0.5 mg	2, 4, 6, and 12
*Bacillus* sp.	1.0 mg	2, 4, 6, and 12
The association of *Pseudomonas fluorescens + Micrococcus* sp. *+ Flavobacterium* sp. *+ Bacillus* sp.	0.2 mg 0.5 mg 1.0 mg	2, 4, 6, and 12
Control: mineral medium without microbial culture	1.0 mg	2, 4, 6, and 12

At first, the pesticide was added to a pre-sterilized 100-ml Erlenmeyer flask, at a concentration of 10, 25, and 50 μg/ml in acetone. After the evaporation of the acetone, 50 ml of mineral medium was placed in 100-ml Erlenmeyer flasks and the flasks were shaken for 48 h. The concentrations of Aldrin came out 0.2, 0.5, and 1.0 mg ml^−1^, respectively. The medium was inoculated with a suspension of cells from *Bacillus polymyxa*, *Micrococcus* sp., *Flavobacterium* sp., or *Pseudomonas fluorescens grown* on nutrient media for 48 h (upon completion of the logarithmic phase) with a final density of about 2 × 10^8^ CFU (colony-forming units) ml^−1^. The total biomass of each bacterial culture and microorganism consortium added to the mineral medium with a pesticide was 10 ml, pH 7.2, and 25 °C. Another medium without a bacterial suspension inoculation served as a control. Both the inoculated and un-inoculated samples were incubated under intermittent shaking (180–200 rpm) to provide aerobic conditions. After 2, 4, 6, and 12 days, duplicate flasks from the inoculated and un-inoculated samples were withdrawn aseptically and analyzed for pesticide residues by HPLC, after their extraction in hexane (Barceló 1991).

Aldrin concentration used in the model experiment was fitted with the pesticide threshold dose at which the deceleration of soil biota occurred. The maximum permissible concentration (MPC) of Aldrin is equal to 0.01 mg/kg, and the lethal dose (LD50) is equal to 50 mg/kg. Taking these features into account, we used doses of 0.2 mg (20 times higher than the MPC), 0.5 mg (50 times higher than the MPC), and 1.0 mg (100 times higher than the MPC). We suggest that low (0.2 mg) and medium (0.5 mg) concentrations will stimulate the development of pesticide-degrading bacteria in soil. The highest doses (1.0 mg) inhibit the growth of bacteria, but bacteria with a powerful degradation capacity could continue the transformation of the pesticide.

Abiotic factors such as air, water, temperature, and light were basic requirements for degradation experiments. The experiment was run in a temperature-controlled room with a natural light (12∶12 h) and dark photoperiod simulating conditions of heavy shade and with water temperature at 15 or 25 °C (± 0.5 °C). These temperatures were chosen because there are the average stream temperatures in soils around the burials, and within these bounds degradation processes by living organisms will be possible.

Biodegradation (%) was calculated on the basis of the difference between residual Aldrin in treated samples and the un-inoculated controls. The means and standard deviations (*n* − 1) of three replicates were computed using data analysis tools in the software program MS Excel. Means were compared by least significant difference (LSD) tests with statistical significance at *P* = 0Æ05, using the software MSTATC.

## Results and discussion

### Physico-chemical parameters of the studied soils

Assessment data of some selected physico-chemical parameters in soil samples collected from sites located around three dumping plots (Suzak A, Suzak B, and Balykchy) that have long been exposed to pesticide contamination, and uncontaminated sites, are presented in Table 2.

**Table 2:** Environmental site for the collection of soil samples

Geographic position, elevation, and locality	Zone/ecosystem and soil source type	Physical and chemical characteristics of the soils
Dumping plot Suzak A and B: N 40° 59.625′, E 72° 53.796′ 1136 m elevation above sea level; Jalalabad province, Suzak region The dumping plot has an area of about 0.9 ha. It has free access and contains nine separate tranches with a total of 2000–3000 t of pesticides dumped.	The plot is located on the bank of the Kougart River, in the northeastern part of the Fergana Valley. Light sierozem soils: These soil types are formed by vegetation from the ephemeral desert steppes. Parent rocks are loess-like loams. The climate of this zone is continental. In summer, the air temperature can reach up to 43 °С, and in winter down to 25 °С.	Mechanical composition is silt-loam, sandy loam, and loamy loess. Humus content 0.7–1.7% Total nitrogen 0.1–0.14% The soils are calcareous at the surface: CO_2_ in the upper layer is about 2–3% Mechanical fraction, %: 1.0–0.25 mm; 5,34%; 0.25–0.05 mm: 17.5%; 0.05–0.01 mm: 47.73%; 0.01–0.005 mm; 13.7% 0.005–0.001 mm: 12.97%; <0.001 mm 5.32% Soil pH: 8.0 The values of EC indicated that all samples of the soils are non-saline.
Dumping plot Balykchy N 42° 28′, E 76° 11′ 1900 m elevation above sea level; Issyk-Kul province A former pesticide storage location is located in Balykchy city. Seven or more barrels of liquid pesticide are stored in the crumbling buildings, in poor storage conditions.	The plot is located 300 m from the Issyk-Kul Lake. There is a residential area within 200 m of this site, and livestock are sometimes grazed here. Mountain/valley gray-brown desert rocky soils: These soil types are formed under extreme continental desert climate conditions. The surface is composed of rocky and gravelly soils. The climate is dry; rainfall varies within 100–250 mm. The weather here is dominated by constant winds.	Humus content 0.5–1.5% Total nitrogen 0.1–0.14% Mechanical fraction, %: 1.0–0.25 mm: 5.34%; 0.25–0.05 mm: 17.5%; 0.05–0.01 mm: 47.73%; 0.01–0.005 mm: 13.73%; 0.005–0.001 mm: 12.97%; <0.001 mm: 5.32%; 0.001 mm: Soil pH: 7.6 The values of EC indicated that all samples of the soils are non-saline.

The obtained results revealed that the values of physico-chemical parameters in the soil samples were in the range of 7.35–11.01% for MC, 7.64–8.0 for pH, 0.7–1.7% for humus, and 0.1–0.14% for N. CO_2_ in the upper layer is about 2–3%, and the values of EC indicated that all samples of the soils are non-saline. The soil type in Suzak A and Suzak B is light sierozem, while in Balykchy the soil type is mountain/valley gray-brown desert rocky.

### Pesticide residues in the soil of dumping zones

Toxic, well-known, persistent organic pollutants were identified by chromatography analyses in the soil samples (mg/kg dry weight) (Tables 3, 4, and 5). Many of these compounds are also among the first POPs that were classified, including alpha-HCH, beta-HCH, Aldrin, and others, the use and production of which were to be reduced or eliminated according to the Stockholm Convention from the United Nations Environment Programme (Stockholm Convention 2001). The concentrations of identified compounds in the investigated places theoretically exceeded many times over the threshold dose effective on the biological organisms of the environment. The presented results indicate that the POP pesticide dump sites and their surroundings have areas of high-risk exposure. These pesticides are persistent and contribute to the biological, photolytic, and chemical degradation of the environment, due to their remarkably long half-lives (estimated from months to centuries or even longer). As a result, all humans, fish, birds, and mammals carry a burden of POPs, mainly in their fat tissue (Weber et al. 2011).

**Table 3:** Concentrations of toxic compounds detected in the soil samples from around the Suzak A dumping zone by chemical analysis (Method of external std. 201_13_07_2015-ECD)

Soil sample number	Quantity (mg/kg)	Compound
1	13.801 ± 1.07	Alpha-HCH
2	52.740 ± 1.75	Beta-BHC
3	19.079 ± 1.12	Gamma-BHC
4	52.661 ± 1.69	BHC delta
5	4.537 ± 0.97	Heptachlor
6	7.353 ± 1.02	Aldrin
7	18.000 ± 1.23	Heptachlor epoxide
8	12.122 ± 1.51	Alpha-endosulfan
9	27.567 ± 1.87	4,4′-DDE (13C12)
10	17.778 ± 1.21	Dieldrin, HEOD
11	35.621 ± 1.29	4,4′-DDD
12	25.601 ± 1.77	Beta-endosulfan
13	43.469 ± 2.06	Endrin aldehyde
14	73.873 ± 2.04	Endosulfan sulfate

**Table 4:** Concentrations of toxic compounds detected in the soil samples from around the Suzak B dumping zone by chemical analysis (Method of external std. 201_13_07_2015-ECD)

Soil samples number	Quantity (mg/kg)	Compound
1	979. 504 ± 1.23	Alpha-HCH
2	577.502 ± 1.09	Beta-BHC
3	910. 548 ± 1.25	Gamma-BHC
4	2283.103 ± 2.09	BHC delta
5	1371. 921 ± 1.87	Heptachlor
6	1326.939 ± 1.76	Aldrin
7	15,418.160 ± 1.86	Heptachlor epoxide
8	462.358 ± 1.53	Alpha-endosulfan
9	654.768 ± 1.34	4,4′-DDE (13C12)
10	268.031 ± 1.23	Dieldrin, HEOD
11	266.593 ± 1.32	4,4′-DDD
12	743.211 ± 1.15	Beta-endosulfan
13	567.780 ± 1.11	Endosulfan sulfate

**Table 5:** Concentrations of toxicant compounds detected in the soil samples from around the Balykchy dumping zone by chemical analysis (Method of external std. 201_13_07_2015-ECD)

Soil samples number	Quantity (mg/kg)	Compound
1	20.764 ± 1.09	Alpha-HCH
2	4.995 ± 0.98	Beta-BHC
3	7.133 ± 1.16	Gamma-BHC
4	1.813 ± 0.88	BHC delta
5	4.121 ± 1.15	Heptachlor
	23.956 ± 1.56	Heptachlor epoxide
7	5.024 ± 1.17	Alpha-endosulfan
8	4.629 ± 1.18	4,4′-DDE (13C12)
9	5.549 ± 1.20	Dieldrin, HEOD
10	8.836 ± 1.73	4,4′-DDD
11	16.527 ± 1.56	Beta-endosulfan
12	14.379 ± 1.93	Endosulfan sulfate
Accumulated value	1000	

**Fig. 1:**
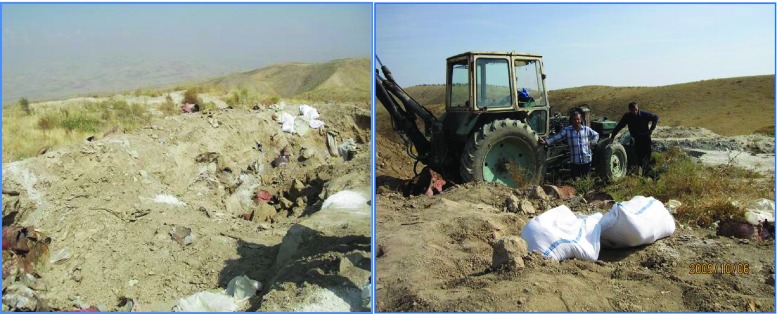
View from dumping plot Suzak A and B (N 40° 59.625′, E 72° 53.796′, 1136 m elevation above sea level), Jalalabad province, Suzak region. Pictured: open pit; people digging out a trench in search of pesticides

For the first time, our chromatographic analysis has revealed the real content of obsolete pesticides in these soils; these results confirm the technical studies of experts (see in particular the Interim Report on the Technical Investigations of Obsolete Pesticides in the Kyrgyz Republic, from April 22, 2010), highlighting that the dumping plots Suzak A and Suzak B are potential “hot spots” by visual inspection. Contaminated for many years, these places are used by the local population as pastures for livestock and sheep. Several times, cases were recorded of a mass poisoning of sheep that grazed around the dumping zones and drank water from the river flowing through these areas. The hazard risk of these places is increased by the fact that local farmers are unaware of the harmfulness and prohibition of these pesticides, and they dig them out of the places they were buried for the purpose of using them in their crops and orchards. Illegal digging further increases the potential for contaminants to enter the environment (Fig. 1).

Thus, in the current study, the chemical analysis of soils around the tailing dumps has revealed the presence of a dangerous source of contamination with high doses of prohibited persistent pesticides in populated areas of the country. It is already well-known that exposure to and accumulation of POPs in the human body have been linked to a wide range of chronic diseases and disorders, like cancer, Alzheimer’s disease, diabetes, and others.

#### Cultivable microbial diversity of contaminated soils

Identified high concentrations of pesticides indicate a need for natural ways to decompose, transform, and eliminate them from the trophic chains of this biogeocenosis. Fortunately, it is known that there are microorganisms that under natural conditions are able to degrade various kinds of pesticides, and they are potentially suitable for use in cleanup techniques and in the design of treatment strategies in polluted ecosystems. The ability to characterize large numbers of microorganisms from the environment by combining phylogenetic, genomic, and biochemical analyses is crucial to developing bioremediation strategies at sites.

Classical microbiological analysis in this study has revealed cultivable forms of microorganisms on different nutrient media. The cultivated method could demonstrate the first microorganisms to grow on contaminated soil as the sole carbon and energy source under aerobic conditions. The poorest microflora was identified in pesticide-contaminated soils, composed of only one or two physiological groups; this indicates that high doses of pesticides inhibit the vital functions of soil microorganisms, and lead to a reduction of their biodiversity in comparison with the biodiversity of uncontaminated soils. The highest count of bacteria in the autumn was found in uncontaminated soil (9.092 CFU/ml). The lowest count of bacteria in the autumn was detected in soil from Suzak A (4.033 CFU/ml). Based on the results, it was observed that there is a significant difference between the soils with and without pesticides at the 5% concentration level.

**Fig. 2:**
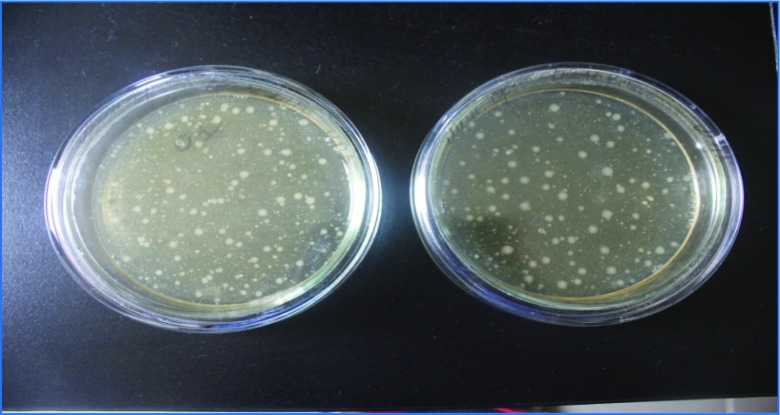
Cultivable microbial diversity of contaminated soils of Balykchy plot on mineral medium: colonies of *Micrococcus*, *Flavobacterium*, and *Pseudomonas* genera bacteria

**Fig. 3:**
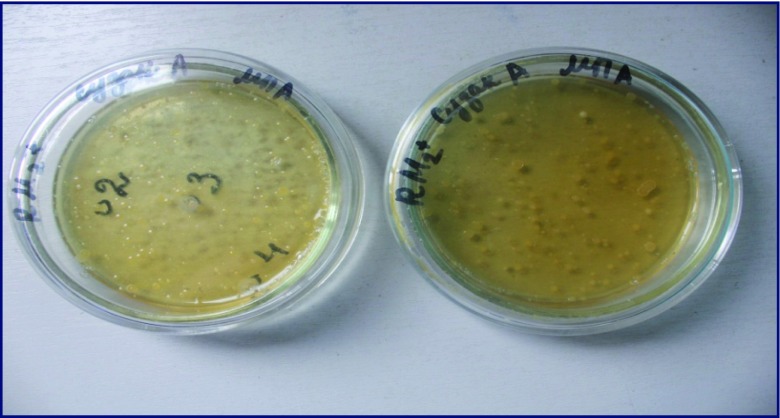
Cultivable microbial diversity of contaminated soils of Suzak plots on mineral medium: colonies of *Micrococcus*, *Bacillus*, and *Pseudomonas* genera

**Fig. 4:**
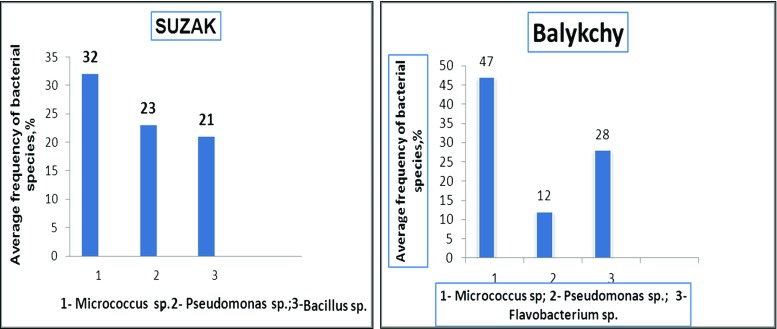
Average frequency of cultivable bacterial species isolated from the soil of dumping zones (Suzak and Balykchy)

Only species resistant to high concentrations of pesticides were detected by cultivation of contaminated soil on the nutrient medium. For example, in the light gray soil from the Suzak plot and from mountain-valley gray soil from the Balykchy plot, bacteria from the *Actinobacteria* phylum were the dominant species. In particular, bacteria species of the *Micrococcus* genus were predominant in soils from around the Suzak and Balykchy dumping places (Figs. 2 and 3). These bacteria made up 32.0 ± 0.07–47.0 ± 0.12% of the indigenous local microflora; bacteria species from the *Pseudomonas* genus (*Gammaproteobacteria* phylum) constituted 23.0 ± 0.07% of the indigenous local microflora in Suzak and 12.0 ± 0.35% of the indigenous local microflora Balykchy soil. *Bacillus* species bacteria were found only in Suzak soil, and their content constituted between 21.0 ± 0.09 and 71.0 ± 0.89% of the indigenous local microflora. Bacteria of *Flavobacterium* sp. were obtained only in Balykchy soil (Fig. 4). These bacteria species, isolated from heavily contaminated soils, were then screened for their ability to degrade Aldrin.

**Fig. 5:**
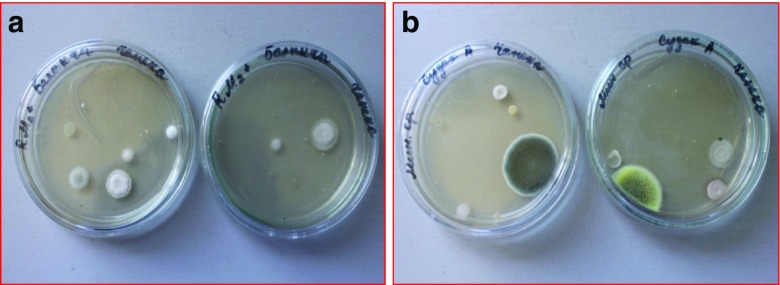
Cultivable diversity of fungi of contaminated soils (on mineral medium). **a** Colonies of fungi found in Balykchy plot soils (*Botrytis*, *Phialophora*, and *Plectosphaerella* genera). **b** Colonies of fungi found in Suzak plot soils (*Penicillium* and *Acremonium* genera)

**Fig. 6:**
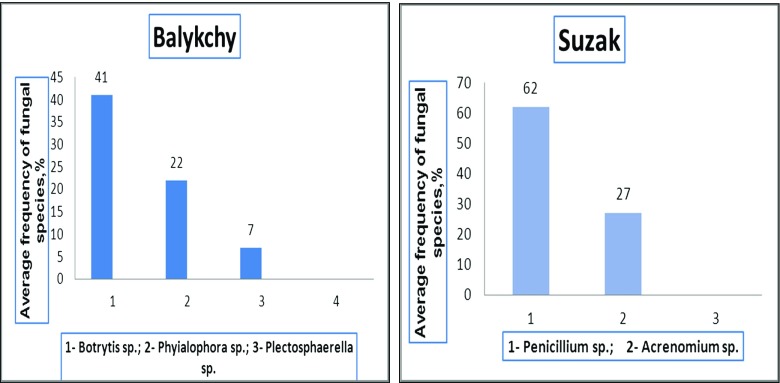
Average frequency of cultivable fungal species isolated from soil of dumping zones (Balykchy and Suzak)

Fungi as representatives of the soil microflora were found in both soils. In Balykchy plot soils, fungi from the *Botrytis*, *Phialophora*, and *Plectosphaerella* genera dominated*.* In Suzak plot soils, fungi from the *Penicillium* and *Acremonium* genera were found (Figs. 5 and 6).

### Biodiversity of uncultured bacteria isolated from contaminated soils by PCR analysis

**Fig. 7:**
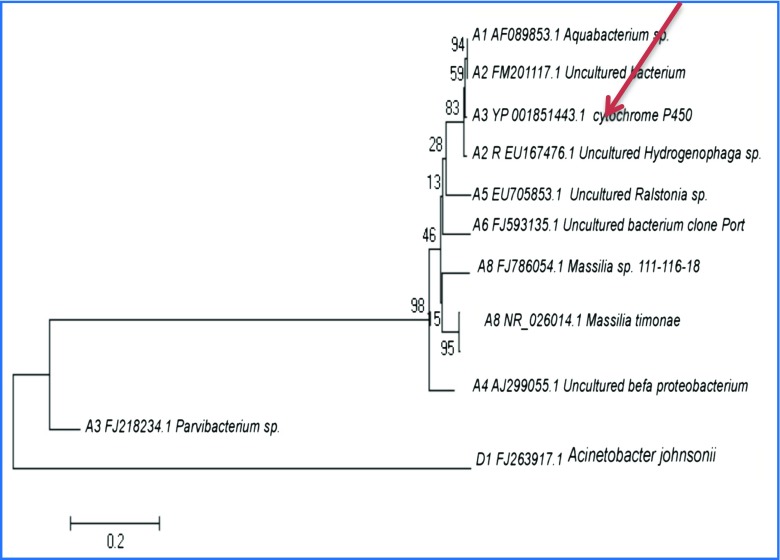
Phylogenetic tree based on 16S rRNA gene sequences showing the position of isolated bacteria strains from contaminated sites

PCR analysis of contaminated soils has enabled the detection of uncultivable soil microorganisms. The 16S rRNA gene sequences taken from uncultured soil samples confirmed that most of the bacteria belonged to classes *Beta, Alpha* and *Gamma Proteobacteria* and *Firmicutes*. Representatives of the *Actinobacteria, Firmicutes* and *Gammaproteobacteria* phyla were predominant in contaminated soils, whose species are active degrading of xenobiotics in nature (Table 6). Among uncultivable forms, the 16S rRNA analyses have found the bacteria with cytochrome genes which are directly involved in the degradation process of organic carbon compounds (Kim et al. 2002). The specific primers used in this study have allowed us to detect the P450 genes that are responsible for degradation activity in bacterial metabolisms, and the results have indicated that the natural pesticide biodegradation process in these soils is conducted by the local microflora (Fig. 7).

**Table 6:** Percentages of bacterial taxa detected in pesticide-contaminated and uncontaminated soil samples

Groups	Contaminated soils		Uncontaminated soils	
	Cultivable isolates (%)	16S rRNA sequence (%)	Cultivable isolates (%)	16S rRNA sequence (%)
*Alphaproteobacteria*	–	1.3 ± 0.01	5.1 ± 0.03	–
*Firmicutes*	30.8 ± 0.21	47.2 ± 0.21	1.2 ± 0.01	10.2 ± 0.54
*Gammaproteobacteria*	3.9 ± 0.11	32.8 ± 0.15	–	12.0–15 ± 0.74
*Betaproteobacteria*	–	1.0–1.4 ± 0.06	–	28.5 ± 0.91
*Bacteroidetes*	0.5–0.7 ± 0.03	1.0–1.2 ± 0.03	7.0 ± 0.61	12.0–17 ± 0.81
*Actinobacteria*	50.3 ± 0.83	47.3 ± 0.96	12.8 ± 0.16	14.2 ± 0.72

The taxonomic affiliation of bacteria was determined by BLAST and by neighbor-joining analysis

Pesticide contamination seemed to facilitate the propagation of *Actinobacteria*, *Firmicutes*, and *Gammaproteobacteria*; these bacterial groups are known to be commonly associated with the hydrocarbon degradation processes. Reported microbiodegraders, obtained by researchers from different countries, belong to the following bacterial classes: gamma-proteobacteria (e.g., *Pseudomonas*, *Aerobacter*, *Acinetobacter*, *Moraxella*, *Plesiomonas*), beta-proteobacteria (e.g., *Burkholderia*, *Neisseria*), alpha-proteobacteria (e.g., *Sphingomonas*), actinobacteria (*Micrococcus*), and *Flavobacteria* (*Flavobacterium*) (Chaillan et al. 2004; Haddock and Gibson 1995). Indeed, bacteria related to the *Pseudomona*s, *Neisseria*, *Moraxella*, and *Acinetobacter* genera are able to degrade the pesticide DDT almost completely (Carrillo-Pérez et al. 2004), while microorganisms from the *Pseudomonas*, *Bacillus*, *Trichoderma*, *Aerobacter*, *Muchor*, *Micrococcus*, and *Burkholderia* genera have been shown to biodegrade dieldrin and endrin (Matsumoto et al. 2009; Hayatsu et al. 2000; Matsumoto et al. 2008).

**Fig. 8:**
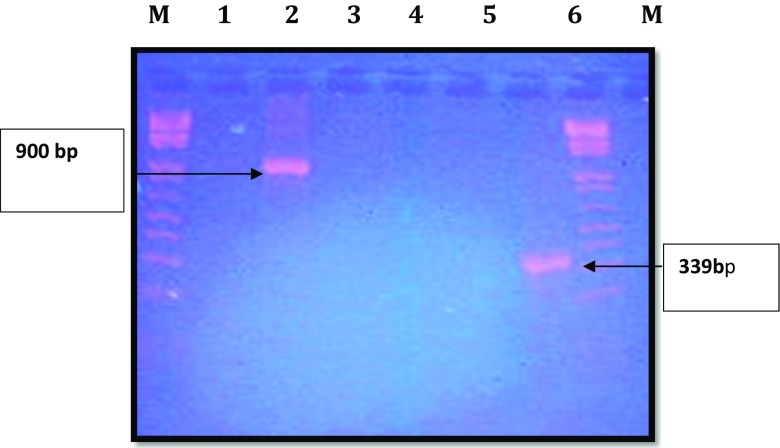
Agarose gel (1.0%) electrophoresis of some PCR products of DNA samples extracted from enrichment culture obtained with universal and specific primers: lanes M—molecular weight markers; lanes 1, 3, and 5—negative controls; lane 2—DNA sample (A-3) amplified with 16Sp RNA gene; lane 4—DNA sample (A-3) not amplified with alkane hydroxylase genes (alkB-F and alkB-R); lane 6—DNA sample (A-3) amplified with cytochrome P450 hydroxylase genes (P450F and P450R)

Most of the DNA samples extracted from the soil in the enrichment culture were amplified with P450 fw1 and P450 rv3 primers, demonstrating the presence of cytochrome P450 genes (Fig. 8). This indicates the presence of pesticide-degrading bacteria genes, which catalyze the degradation pathways of the organochloride compounds in these soils.

### Biodegradation experiments

The microbial degradation of pesticides, a process known as bioremediation, is a cost-effective method of removing pollutants from the environment. To optimize the conditions, it serves as an important parameter for ensuring complete biodegradation and bioremediation of polycyclic aromatic hydrocarbons (PAHs) on site. Working conditions, namely, substrate concentration, bacteria concentration, pH, and temperature, have to be optimized. Results from the optimization study of biodegradation indicated that the maximum rate of PAH removal occurred at 100 mg L^−1^ of PAHs, 10% bacteria concentration, pH 7.0, and 30 °C (Othman et al. 2016). For the degradation of pesticides, bacteria must possess enzymes that cleave the ring of the aromatic halogen compound (PCP) (Arora et al. 2010; Arora and Bae 2014; Zhou et al. 2008).

Aldrin is an organochlorine insecticide that was widely used until the 1970s, when it was banned in most countries (Chemicals Regulation Directorate 2009). It is a colorless solid. Before the ban, it was heavily used as a pesticide to treat both seeds and soil (Robert 2002). An estimated 270 million kilograms of Aldrin and related cyclodiene pesticides were produced between 1946 and 1976. In soil, on plant surfaces, or in the digestive tracts of insects, Aldrin oxidizes to the epoxide dieldrin, which is more strongly insecticidal. Like related polychlorinated pesticides, Aldrin is highly lipophilic. Its solubility in water is only 0.027 mg/l, which exacerbates its persistence in the environment (Centers for Disease Control and Prevention 2011). Aldrin seems more persistent in respect to biodegradation as compared to lindane (gamma-HCH) and chlordane, for example (Kennedy et al. 1990). In Kyrgyzstan, this insecticide was widely and intensively applied against pests of cotton and other crops until the 1980s. It was detected at high concentrations in all the investigated dumping zones. We decided in our model experiments to test bacteria that were isolated from pesticide-contaminated soils on their ability to degrade Aldrin in different concentrations. The maximum permissible concentration (MPC) of Aldrin is equal to 0.01 mg/kg, and the lethal dose (LD50) is equal to 50 mg/kg. Taking into account these features, we used doses of 0.2 mg; this is 20 times higher than MPC, and 0.5 mg is 50 times exceeding the MPC and 1.0 mg (100 times higher than the MPC). We suggest that low (0.2 mg) and medium (0.5 mg) concentrations will stimulate the development of pesticide-degrading bacteria in soil. The highest doses (1.0 mg) inhibit the growth of bacteria, but bacteria with a powerful degradation capacity could continue the transformation of the pesticide.

**Fig. 9:**
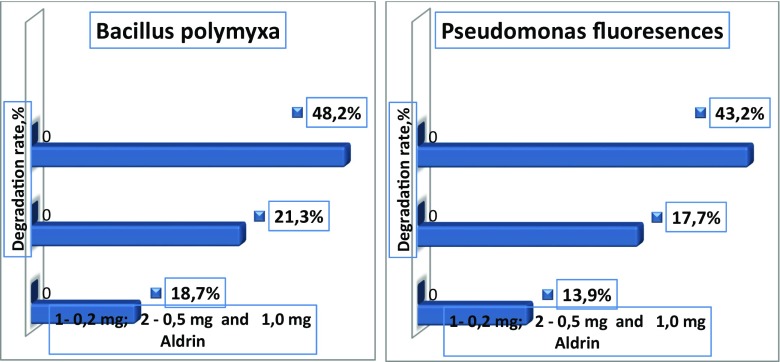
Degradation rate of Aldrin by single cultures of *Bacillus polymyxa* and *Pseudomonas fluorescens* in mineral media over 12 days

**Fig. 10:**
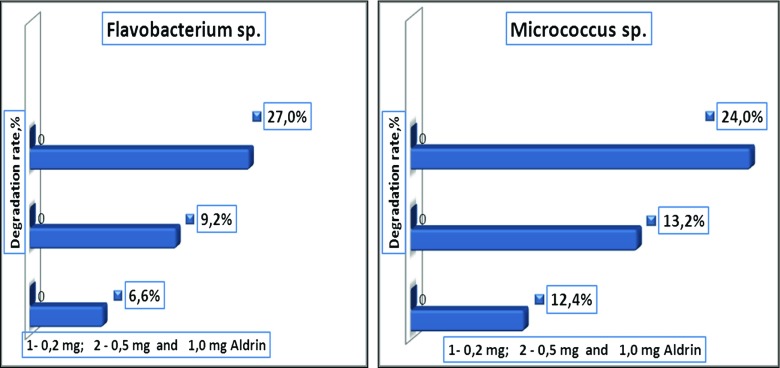
Degradation rate of Aldrin by single cultures of *Flavobacterium* sp. and *Micrococcus* sp*.* in mineral media over 12 days

**Fig. 11:**
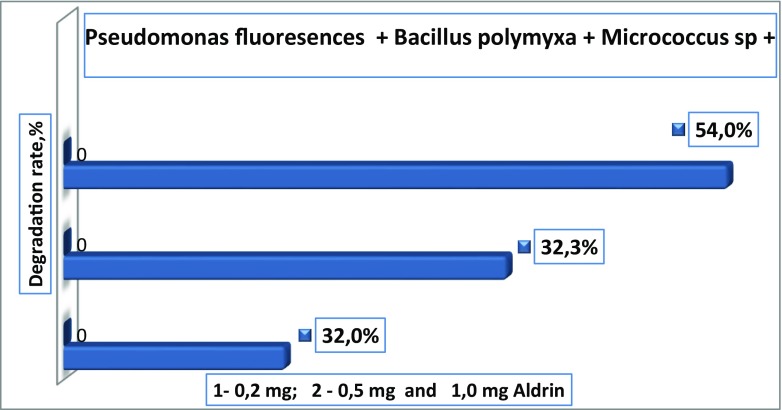
Degradation rate of Aldrin by *Pseudomonas fluorescens* + *Bacillus polymyxa + Micrococcus* sp. *+ Flavobacterium* sp. in association, in a mineral medium over 12 days

Results have shown that Aldrin was degraded by bacteria at different levels. The bacterium with the highest capacity to degrade Aldrin was *Bacillus polymyxa* in single cultures. This bacterium used in its metabolism 0.2 mg of Aldrin and reduced its content to 48.2% after 12 days of incubation. *Pseudomonas fluorescens* has also shown a good degradation capability. This bacterium degraded 43.2% of 0.2 mg Aldrin. *Flavobacterium* sp. and *Micrococcus* sp. bacteria have shown a minimal degrading capability. These bacteria degraded 27.0 and 24.2% of 0.2 mg Aldrin, respectively (Figs. 9 and 10). The best degradation capacity of Aldrin at the 0.2 mg concentration occurred in the association of these bacteria after 12 days of incubation (Fig. 11). These results indicate that the 0.2-mg doses of Aldrin (this is 20 times higher than MPC) can be rapidly destroyed by bacteria with high degradation capabilities in a relatively short period of time.

**Fig. 12:**
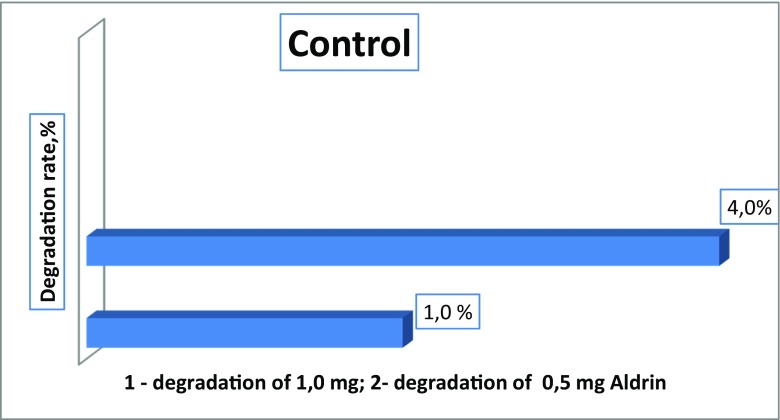
Degradation of Aldrin in control

Higher doses (0, 5, and 1.0 mg) of Aldrin were destroyed slowly, and the rates of degradation by bacteria were not high (32.3, 21.3, 18.7, 17.7, and 13.9%, depending on bacteria species). However, it should be noted that even high doses that exceed the permissible concentrations several times over can be destroyed and transformed by bacteria isolated in these contaminated areas. In this model experiment, Aldrin at concentrations of 0.5 and 1.0 mg was degraded more intensively by associations of bacteria than by single cultures. In the control, in which Aldrin was maintained in the mineral medium for 12 days with constant shaking and without bacteria cultures, there was a minimal reduction in the amount of Aldrin by 1.0% at a dose of 1.0 mg, and by 4.0% at a dose of 0.5 mg. Apparently, there was a purely mechanical and physical removal process (for example, by evaporation) of the pesticide (Fig. 12).

With further continuation of these experiments, these bacteria will eliminate these high concentrations of contamination under appropriate abiotic conditions after a certain period of time. In these experiments, the important abiotic parameters were aeration, temperature, humidity, and light, such that conditions close to natural conditions were created for full-fledged work of aerobic selected degrading bacteria. Such conditions occur naturally in the spring, autumn, and summer, with rain. For intensive acceleration of this process, it is necessary to add bio-preparations based on selected active destructors.

It is consequently possible that the degrading capacity of the bacteria could be increased only through co-cultivation, which shows that these bacteria naturally coexist and are dependent on each other for the utilization of environmental substances. In the oxidation and hydrolysis pathways of pesticide degradation, each bacterium can produce metabolites that will be utilized by the enzyme system of the next bacterium.

Some researchers have noted two further factors worth mentioning: co-metabolism and consortia conditions. Some biodegraders require other substrates in order to degrade pollutants (Alexander 1999). This phenomenon is called co-metabolism and is especially necessary for organochlorine compounds. In contrast, it has been shown that the presence of other carbon sources decreases the rate of organophosphate biodegradation (Hayatsu et al. 2000).

When pesticide degradation occurs, it usually involves more than one microorganism, i.e., each microorganism contributes to the biodegradation pathways of the pesticides, while no example of mineralization by a single strain has been reported. It seems that the presence of different microorganisms is essential for adequate biodegradation to occur (Velázquez-Fernández et al. 2012; Romeh and Hendawi 2014).

Despite the fact that Aldrin has low solubility in water, the bacteria used in this study have shown their ability to break down the structure of this substance, turning Aldrin into simpler compounds and ultimately to the final products of water and carbon dioxide. This is confirmed by decreasing the amount of this pesticide added to the mineral medium, where bacteria cultures were grown.

## Conclusions

Soils contaminated with pesticides have attracted significant attention because soil contamination impacts human health and the natural ecosystem. Microorganisms that are present in the soil can remove pesticides from the environment.

Ecosystems in Kyrgyzstan contaminated by obsolete pesticides need urgent attention, and require solutions to avoid further contamination that would affect a population’s health. Our results have revealed the occurrence of a natural biodegradation process of pesticides by local soil microorganisms in soil samples from around the Suzak A, Suzak B, and Balykchy dumping zones.

The presence of such microorganisms involved in the biodegradation process was proved by amplification of the cytochrome P450 genes that encode this process. The bacteria with the cytochrome genes that are directly involved in the degradation process of the organochloride compounds were selected for model experiments in order to catalyze the degradation pathways of the POP pesticide, Aldrin, which was found in the studied soils in high concentrations. Active *Pseudomonas fluorescens* and *Bacillus polymyxa* bacterial strains were selected for this research. They have demonstrated high rates of degradation activity on the chlorinated hydrocarbon pesticide Aldrin in a relatively short time (12 days), both in association and in single cultures. These bacteria should be recommended for external addition to soil where innate microbial populations are not able to manage pesticides. Degradation of pesticides by microbes depends not only on the enzyme system but also on the conditions like temperature, pH, and nutrients. The efficiency of the degradation process depends upon optimum atmospheric conditions, that is, temperature, pH of soil, moisture contents, and so forth. The biodegradable removal of pesticides has positive effects on the fertility of agricultural soil too.

Further research will include other pesticides and pesticide mixtures present at the sites, and will optimize the pesticide degradation conditions in a variety of soil types, using the bacterial strains selected for this study. It will be necessary to optimize the physical and chemical parameters, taking into account the type of pesticide and the metabolism of the microorganism itself and to monitor and facilitate pesticide degradation at and around the dump sites.
